# A Practical Treatise on the Diseases of Children

**Published:** 1848

**Authors:** 


### Part 2.—Reviews and Notices of New Works.

### ARTICLE I.

*A Practical Treatise on the Diseases of Children.* By J. FORSYTH MEIGS, M.D., &c. Philadelphia: Lindsay & Blakiston. 1848. pp. 575 8vo.

*Sage enfant qui connoit son père*, says the old French proverb, and in these book-making days it may be not inaptly said that it is a wise volume that can trace its own pedigree. Originality is a commodity that is well nigh obsolete, and in lieu of it we have compilation better or worse. Especially is this the case in Medical Science; and the fact has become so notorious, that physicians perhaps read less than any other class of professional men, confining their efforts in the way of reading, principally to the short essays and reports furnished in the medical periodicals of the day. We are fully aware that in the above remark upon originality, we have advanced nothing new; even so far back as the days of Chaucer, to say nothing of Solomon, the same idea was entertained.

> "Out of the olde fieldes, as men saithe,
> Cometh all this new corn fro yere to yere;
> And out of olde bookes in goode faithe,
> Cometh all this new science that men lere."

Its rare to find, even in Medical Journals, an analysis of the various works from time to time issued by our enterprising publishers: we have merely a short notice—nine times out of ten entirely approbatory—containing the title, name of the author, a hasty and imperfect sketch of the table of contents, and perhaps a page or two by way of extract. Medical editors evidently esteem it too great a bore to examine attentively all the works which are furnished them by the courtesy of authors and publishers; and as good manners forbid an entire disregard of such favors, a random compliment is inserted; but as to anything like a searching analysis, or examination of the doctrines advanced, the thing is not to be thought of. The fact is, our medical journals are somewhat faulty in this particular: physicians look to them for all news interesting to the profession, and the meager notices of books which are furnished are not sufficient to inform a physician distant from the great cities and thoroughfares, whether or not a given volume is worthy a place in his library. In consideration of this defect in the literature of our profession, we promised in our last number, to give a somewhat extended notice to the work whose title stands at the head of the present article.

It is a generally received opinion amongst mankind, that experience is necessary to excellence in any art or profession, though this opinion is often carried to a ridiculous extent, by persons who forget that talent and close observation are essential to render experience of any value. Our author has cut loose from this long established dogma, and although a junior member of the profession, has written a treatise for the instruction of both students and practitioners; and we cannot say that the task is illy executed, or that the work is a useless addition to our already extensive literature. The author has availed himself of the opportunity of consulting various English and French authorities, the latter of which, are not generally accessible, owing to the fact of their being written in a foreign language; and has collected from various medical journals many items of interest and value, which would otherwise be lost to the majority of the members of the profession. The details of his personal experience are not extensive, but we suppose they are in the main accurate. We desire, also, to bear testimony to the excellent mechanical character of the work: the paper is good, the type clear, and there is a singular freedom from typographical blunders; moreover, the price is so low as to place the work within the reach of all.

All diseases of children are classified under five general heads, viz: Diseases of the Respiratory Apparatus, Diseases of the Digestive Apparatus, Diseases of the Nervous System, Eruptive Fevers, and Worms in the Alimentary Canal. Class I. is divided into two chapters, the first containing a description of diseases of the upper air passages, and the second, diseases of the lungs and pleuræ. Chapter 1 treats of coryza, pseudo-membranous laryngitis, spasmodic laryngitis, and simple laryngitis. Coryza being a simple disease, and very rarely dangerous, need not detain us. Concerning the section on croup, we have somewhat to say. In the first place, we object to the nomenclature employed by Dr. M., as radically faulty, and tending to convey a false notion of the pathology of the disease under consideration. Pseudo-membranous laryngitis, would seem to designate a complaint affecting exclusively or principally the larynx, and attended with, or marked by, the exudation of false membrane. Now that croup has its principal seat in the larynx, we are by no means disposed to admit, and in support of our assertion we have the weight of modern authority. Stewart, Dewees, Evanson and Maunsel, Elliotson, Watson and Williams, regard the trachea as being the organ most frequently implicated; Condie regards croup as a laryngeo-tracheitis; Bell seems to regard it as primarily affecting the larynx, and the same view is taken by Bichat, and John Mason Goode, the latter of whom, seems to have very indefinite notions of the disease. Dr. M. himself, admits that, in, perhaps, two thirds of the cases, the larynx and trachea are both involved. Now, in nosology, as in any other branch of science, a name should designate something of the nature of what it stands for; and although tracheitis may be an objectionable term for croup, we conceive that pseudo-membranous laryngitis is much more so. Were croup principally a disease of the larynx, we might hope great things from tracheotomy, against which, the weight of British and American authority is decided, and in our opinion conclusive. Should Dr. M. still see fit to object to the old name of tracheitis, we would commend to his notice the term used by Dr.

Condie, as being nearer the mark than the tremendous cognomen he has seen fit to employ. We object to regarding pseudo-membranous croup, and spasmodic croup, as two separate and distinct affections: we deny that the fact of the exudation of false membrane is sufficient to distinguish one inflammation from another, or that the spasmodic action of the muscles—unless such spasm is totally independent of other pathological conditions—is sufficient to authorize a writer to introduce a new species into nosology. In opthalmia it is a common thing to see effusion of lymph into the anterior chamber of the eye (now the false membrane of croup is nothing more than organized lymph); but we apprehend that an author would be considered as given to unnecessary refinement, who would designate those cases accompanied with effusion as constituting a disease distinct from ordinary opthalmia. It seems to us, that what our author designates as pseudo-membranous laryngitis is a severe form of croup, and the spasmodic variety, the same disease in a milder form. As an author, writing in an age when most subjects of interest have been fully discussed by the ablest members of the profession, Dr. M. may be anxious to present something that shall bear the impress of novelty and originality; but he should not forget, that in our profession he renders a service who simplifies what has heretofore been complicated, and that the improvements in medicine and surgery have generally tended in this direction. Now the praise of simplicity and conciseness is something we cannot award to the technology of the article on croup. That part which refers to the treatment of this severe and dangerous complaint, we believe to be judicious. One article is recommended as an emetic, which, so far as we know, is not in general use amongst us: we refer to the alum; the advantages of this remedy are thus set forth by the author:

The alum is given in powder, in the dose of a teaspoonful, mixed in honey or syrup, to be repeated every ten or fifteen minutes until it operates. It is very seldom necessary to give a second dose, as one operates in the majority of cases very soon after being taken. I have known it to fail to produce vomiting only in two instances, both of which were fatal cases. In one the disease had gone so far before I was called, that no remedy had any effect upon the stomach. In the other, it was administered several times with full success, but lost its effect at last, as had happened also in regard to antimony and ipecacuanha. The reasons for which I prefer alum to antimony, or ipecacuanha, are the following; antimony, when resorted to as frequently in the disease as I am of opinion that emetics ought to be, is too violent in its action; it prostrates many children to a dangerous degree, and is, I fear, in some cases, itself one cause of death. It acts injuriously upon the gastro-intestinal mucous membrane, when used in large quantities, and for any length of time. Again, it is very apt to lose its effects and produce sickness. Ipecacuanha is a much safer remedy than tartar emetic, but its operation is often too mild, and it also ceases to produce any effect after it has been used several times. The advantages of the alum are, that it is certain and rapid in its action, and that it operates without producing exhaustion or prostration beyond that which always follows the mere act of vomiting. It does not tend like antimony, and in a less degree ipecacuanha, to produce adynamia of the nervous system; an effect which, in some constitutions or states of the constitution, or when it has been exhibited frequently, is often attended with injurious or even dangerous consequences. I have given alum in the dose above mentioned, twice and three times a day, for two and three days, without observing any bad effects to result from it. The alum was given in all the cases that I have seen, in which emetics were used, and was the only one employed when it was found to produce full vomiting, with a single exception, one of the cases accompanied by violent angina, in which ipecacuanha was substituted because of its smaller bulk. I have already said that it failed to produced vomiting only in two instances. It was the emetic employed in the three cases in which fragments of false membrane were rejected, and in that in which the yellow viscid fibrin was expelled. Although it did not occasion the rejection of membrane in the other cases, it operated most speedily and efficiently.

While on this part of the subject we will take occasion to make a remark or two on the literary character of the work. It is a notorious fact that the technology of the medical profession is peculiarly barbarous: this is, perhaps, owing in a great degree, to the fact, that the ancient works on medicine were put forth in the dead languages, and that up to a comparatively recent period, the Latin language was the universal medium of communication amongst the learned; and it was natural to suppose that many terms derived from these languages would still be retained; but books are now written in the vernacular language of the author, and it should be the aim of every writer to use as plain and simple language as the nature of the subject will admit, and as few technicalities as possible. It is true that it would be an advantage to all physicians to obtain a liberal education, and especially to be familiar with those noble languages, the Latin and Greek; but in the United States, and especially the Western States there are many reputable practitioners who know little of any language save their mother tongue, and the wants and capabilities of this class should be considered. But, taking the thing on broader grounds, it is stated by Lord Kames as a rule in criticism, that no one is authorized to introduce a new term, unless there be no word in the vernacular that will equally well convey a correct and definite idea of the thing to be described. Now we suggest that *foot-bath* is quite as definite, expressive and euphonious a term as *pediluvium*, and so of *manuluvium*: following our author's fancy for magniloquence, we suppose hip-bath would be *coxæluvium* and a bath proper, a *totiluvium*. Every Greek scholar knows that *prodromus* is synonymous with *precursor*, but we doubt whether this is a fact generally known. The fact is, we think the employment of such terms smacks of pedantry, a something which we thoroughly dislike, and, although we do not hold Dr. M. accountable as the inventor of such terms, we must hold him, as the lawyers say, *particeps criminis*.

We pass from the consideration of croup to bronchitis; and here again we are free to commend all our author has put forth, save his nosology. Here we have "*acute bronchitis* of moderate severity," *acute suffocative bronchitis*, and sub-acute or chronic bronchitis. We confess ourselves unable to see the necessity for the distinction drawn between the first two forms of this disease; it would seem to us as well to call it mild bronchitis, and severe bronchitis; and following our author's taste for minute sub-division, we could make out more species of fever than Cullen ever dreamed of, if every accidental complication were to be considered as constituting a distinct disease. No system of nosology can be formed that would correctly indicate to a practitioner the course of treatment to be pursued; unless he be one of those who go through a routine sort of practice: any accidental complication produced by constitutional peculiarities or other causes, must be left to the well informed judgment of the attending physician; and if he have not the ability to provide against such difficulties as they arise, no nosology however skillful will help him out of his trouble.

The article on pneumonia of children is prepared with great care, and is doubtless a highly valuable treatise on a disease the peculiarities and complications of which, many of our physicians have not sufficiently studied. The distinctions of different conditions of the lungs can be drawn with greater clearness than those of other organs of the system, and yet we have known many passing for respectable practitioners of medicine, who possessed a very scanty knowledge of the science of auscultation. Towards the close of the remarks on pneumonia, we are presented with a discussion on the use of tartarized antimony. Our author condemns the common mode of exhibiting this energetic therapeutic agent. He thinks physicians are in the habit of using this drug in too large quantities. There is no doubt that in children the mucous membrane is often extremely sensitive, and there is danger that gastric irritation may be excited by an incautious use of this remedy; but from a pretty extensive experience we are inclined to suppose that the danger is not usually so great as he seems to apprehend. We have used this drug pretty freely in pneumonia, bronchitis, and those cases of measles in which the stress of the disease fell upon the respiratory apparatus; and, in reviewing our practice, we see little to regret as to the use of tartar emetic. We think if a physician will watch his cases closely, there is little danger of any severe mucous derangement occurring; and we are free to confess that we would be at a loss in treating ordinary pneumonia or bronchitis were we to be deprived of this drug which possesses such powerful control over the heart's action; nor can we easily believe that from half a grain to a grain in twenty-four hours, administered to children from two to four years of age, would produce much impression on a disease so severe in its character, and rapid in its progress as pneumonia. Nevertheless, we concur in recommending to the young practitioner a proper degree of care and watchfulness in the administration of all such powerful therapeutic agents as antimony. All physicians who have had extensive experience in the treatment of pneumonia as it occurs in children, must have met with cases in which the diagnosis was a matter of difficulty. The following cases are instructive examples of the insidious forms which this disease sometimes assumes:

I may mention, in illustration, that I attended a boy six years old, who, for three days, suffered from violent fever, and excruciating headache, which last was the only symptom complained of. There was neither cough, expectoration, nor any marked acceleration of the respiration. After three days the headache moderated, and he had slight pain in his side; on examination, I found him laboring under well-marked lobar pneumonia. Another child, four months old, was suddenly seized with convulsions, followed by fever, vomiting, excessive irritability and drowsiness, so that I supposed the case to be one of meningitis. After the third day, the cerebral symptoms having moderated, and cough, with dyspnœa, making its appearance, I detected the existence of extensive lobular pneumonia, of which the child died a few days after. In April, 1847, I was called to see a boy nineteen months old, who had been taken sick with slight fever, a little hoarse cough, and mild pharyngitis. After remaining in this condition for five days, he began to be drowsy and very irritable; the surface became pale, and the extremities rather cooler than natural. From the sixth to the tenth day, there was great somnolence, the child sleeping nearly all the time; when waked from sleep, he was always exceedingly irritable and cross, scarcely opening his eyes, and then shutting them again immediately, to avoid the light, which was evidently painful. During this time he took scarcely any food, but little drink, and vomited several times freely; the bowels were moved without medicine; the surface remained very pale, and the extremities often cool; the pulse was frequent and small; the respiration perfectly regular, and therefore attracted no attention; there was *no cough* whatever. Under these circumstances, I hesitated between regarding the case as meningitis, or hydrocephaloid disease, as described by Dr. M. Hall. I took the latter view, however, and treated it with small quantities of brandy, cold to the head, and the frequent employment of mustard pediluvia. From the eleventh day the child began to improve; it would open its eyes from time to time, and look round for a few moments; the face began to show a slight degree or color, and the palms of the hands which had been white and transparent, exhibited a tinge of the natural pink hue which they have in children. Observing about this time that the respiration was accelerated though perfectly free and regular, and without cough, I counted it, and was astonished to find it 80 in the minute. I now examined the chest carefully, and finding slight dulness on percussion with bronchial respiration, over the inferior half of the left side behind, immediately understood the nature of the case: it was one of latent pneumonia, simulating hydrocephalus. The child was now treated for pneumonia, and after an illness of twenty-seven days longer, recovered perfectly. As the case progressed, the rational signs of pneumonia were more and more apparent, the cough becoming frequent and painful, and after a time loose, while the cerebral symptoms gradually disappeared.

In addition to these cases I have met with three others, two in children within the year, and one in a child between one and two years old, which, during the early stages, resembled very closely the invasion of cerebral disease. Attention, however, to the rate of the respiration and the physical signs, and the presence of slight cough in two of them, revealed, after a little hesitation, the true character of the attacks. The third case, which occured in the child within the year, was unattended by any cough during the first few days, and was, therefore, very obscure, until my attention was attracted by an acceleration of the respiration, when the physical signs, and at a later period, cough, explained the real nature of the attack. I may remark, in addition, that in all these cases, the absence of constipation, the infrequency and short duration of the vomiting, and some clearness of the intelligence when the child was fairly roused, though but for a few moments, from its state of somnolence, were other motives for doubting the attacks to be meningitis.

The portion of the work devoted to pleurisy and hooping cough presents nothing new or peculiar except the use of alum in the latter affection, which remedy we recommend our readers to try. By the way, speaking of hooping cough, we have often thought it singular that our art has done so little towards cutting short the career of this troublesome complaint. Perhaps the very fact that it is very seldom dangerous, has led physicians to bestow very little attention upon it.

We come, in the next place, to speak of what we consider by far the best part of the volume: we refer to the division treating of diseases of the digestive organs, and we apprehend that the reason of the superiority of this section is owing to the author having had more experience in the treatment of diseases of these organs than in any others. It is in medicine as in every thing else—experience alone can give readiness and a decision in the application of principles. In no class of infantile diseases does the physician so much need an intimate acquaintance with various modes of treatment, and the light derived from experience, as in some of the bowel affections of children. We recollect hearing the wife of a distinguished gentleman offer as her reason for employing a homœopathist, what she stated as an acknowledged fact, that the regular physicians could not cure the summer complaint. Now although we do not agree with her in this sweeping denunciation of the faculty, we are free to confess that we have long esteemed cholera infantum as one of the *opprobria medicorum*: but we are anticipating. The diseases of the digestive organs are divided into those of the mouth, throat, and stomach and bowels. We have in the first chapter, enythematous stomatitis, follicular stomatitis, ulcero-membranous stomatitis, stomatitis with curd-like exudation and gangrene—pity that our author had not a word of ten syllables to substitute for gangrene.

In societies where children are raised according to nature, malignant diseases of the mouth are happily infrequent. It is only in crowded cites, unhealthy locations, in hospitals, or when children are deprived, by casualty or design, of the food nature has prepared for them, that we have a great deal to do with dangerous stomatitis, and consequently this chapter has comparatively little interest for us who practice in communities where civilization and her handmaids, vice and poverty, have not attained so great a degree of perfection as in the great cities of Europe and America. We commend this part of our author's work as embodying the results of the observation and experience of the French physicians who, doubtless, have the best opportunities of noting the misfortunes entailed by iniquity upon suffering innocence.

Many of our readers have no doubt been often perplexed by having patients who rejected everything taken into their stomachs, and who were, for this reason, in positive danger of death from inanition. We recommend the following preparation commended by our author, as forming a good combination of animal and vegetable aliment,

I would recommend in these cases a diet which I have found to agree better with children deprived entirely of the breast, than any other that I have ever directed. I have employed it now, in a great many instances, and believe it to be the best substitute for the natural aliment that I am acquainted with. It is made by dissolving a small quantity of prepared gelatine or Russian isinglass in water, to which is added milk, cream, and a little arrow-root, or any other farinaceous substance that may be preferred. The mode of preparation, and the proportions are as follows: A scruple of gelatine (or a piece two inches square of the flat cake in which it is sold) is soaked for a short time in cold water, and then boiled in half a pint of water until it dissolves,—about ten or fifteen minutes. To this is added, with constant stirring, just at the termination of the boiling, the milk and arrow-root, the latter being previously mixed into a paste with a little cold water. After the addition of the milk and arrow-root, and just before the removal from the fire, the cream is poured in, and a moderate quantity of loaf sugar added. The proportions of milk, cream, and arrow-root, must depend on the age and digestive power of the child. For a healthy infant within the month, I usually direct from three to four ounces of milk, half an ounce to an ounce of cream, and a teaspoonful of arrow-root to a half pint of water. For older children, the quantity of milk and cream should be gradually increased to a half or two-thir s milk, and from one to two ounces of cream. I seldom increase the quantity of gelantine or arrow-root.

I have given this food to a great many children for upwards of a year past, as well to those brought up entirely on hand, as those partly suckled, or weaned, and can truly state that they have thriven better upon it than upon anything that I have ever employed. In several cases it has agreed perfectly well with infants who could not, without vomiting, diarhœa, and colic, take plain milk and water, cream and water, any kind of farinaceous food prepared with water, chicken water, or in fact any other food that had been tried. In the cases of sick children, it ought sometimes to be made even weaker for a while, than in the first proportions mentioned above.

We have next, diseases of the digestive tube. These are divided, and we think the division admirable, into functional diseases, and those attended with appreciable anatomical lesions. Upon what pathological condition functional lesions depend, is often a difficult problem to solve. We say that they depend upon irritation of the nervous extremities; but this is not so much a definition, as a mere statement of the question in another form. In this department we have still much to hope from pathological researches. In our author's article on the indigestion of infants, much stress is laid on the quality of the nourishment received by the child from the mother or nurse, and wholesome admonitions are given on this point. He justly ascribes the majority of the cases to improper food, and "bringing up by hand" as it is termed. We have not infrequently seen persons present to infants a piece of fat, rank bacon to suck at; and when afterwards, as was natural to suppose, the little thing rejected the contents of its stomach, or screamed with the colic, the disorder was attributed to the milk souring on the stomach, and a dose of paregoric or Godfrey's cordial, was philosophically sent down to settle the difficuly, and the same course pursued from day to day, until confirmed indigestion was produced. Our author's direction for the treatment of this disorder, we conceive to be highly judicious—we cannot believe with him however, that the diagnosis is so simple as he seems to suppose. Most affections of the bowels in children pass in the community by the designation of summer complaint; and we fancy this is the case occasionally amongst medical men; and that children are *dosed* from day to day with calomel in order to cure a cholera infantum that has no existence save in the imagination of the practitioner. We commend the remarks on the food proper in infantile indigestion. There is no greater humbug than the notion of an exclusively vegetable diet, either for adults or infants. We hold that vegetable, and especially farinaceous food is less adapted to the use of patients suffering from indigestion, than one almost exclusively animal. Milk is as much an animal diet as roast beef, and we cannot but believe that the teachings of nature are safer guides for practice than the speculations of dreamy enthusiasts, for such we must consider the advocates of vegetable diet: but we cannot now discuss a question which would lead us so far from our subject. We take our leave of indigestion with this caution to the young practitioner: when you have a case of bowel complaint to treat, consider well whether you had not better substitute iron, quinine, and port wine for calomel and acetate of lead.

We have next a disquisition upon diarhœa, and a very sensible one. The author discards all the arbitrary and unnecessary divisi ns of this complaint into feculent, bilious, lienteric, etc.; it would be just as useful to and scientific to speak of green apple diarrhœa, and preserved quince diarrhœa. In the treatment of this disease, Dr. M. presents nothing new or of special interest; we therefore pass on to diseases of the intestinal tube, accompanied by appreciable anatomical lesions. The first of these is gastritis: idiopathic gastritis is of rare occurrence in adults, and we presume even less frequent in infants. Under this head is a discussion of the much mooted question whether or not softening of the stomach ever occurs during life: Abercrombie inclines to the opinion that it never does: but, from the opinions cited in this work, we are inclined to think it does.

We come next to speak of a very common and fatal affection of childhood, designated by the author entero-colitis. To show the frequency and fatality of this affection, the following extract is subjoined:

Entero-colitis is one of the most frequent of children's diseases. It appears from a table published by Dr. Condie, (*Dis. of Child.* note, p. 89,) that in the ten years preceding 1845, there were 6068 deaths in Philadelphia under fifteen years of age, from diseases of the digestive organs; of this number 4786 were from diarrhœa, dysentery, cholera infantum, and inflammation of the stomach and intestines, and as entero-colitis exists in by far the greater part of these diseases, we may understand how extremely frequent an affection it is. The deaths from affections of the digestive organs during the period referred to, constituted about a fourth of the whole mortality under fifteen years of age; whilst those from diseases of the brain are stated to have been rather more than a fourth, and from diseases of the respiratory organs nearly a seventh. It appears, indeed, that entero-colitis, in the form of diarrhœa, dysentery, cholera infantum, or what is called in the bills of mortality, inflammation of the stomach and intestines, is by far the most fatal disease of childhood. We may appreciate yet more accurately the importance and frequency of the disease, by reference to the statements of Rilliet and Barthez, who say (t. i, p. 483,) that, taking into consideration all the cases they observed, including tubercular cases, they find that of every two children that die, one presents a more or less serious lesion of the large intestine. They add: "if it be recollected that this holds true particularly in regard to younger children, it will be seen that it is rare for a child to die between two and five years of age, without having either colitis or softening of the large intestine." Bouchut states that entero-colitis is one of the most dangerous affections of children at the breast; "It is the most common of all those incident to that age." (p. 210.)

This disease is very elaborately discussed, and the treatment recommended is in the main judicious; we take occasion, however, to dissent from the author's recommendation as to the use of calomel. We can hardly believe that from one to four grains given in a single dose, and followed up by a laxative, will produce much impression on a violent attack of this complaint. The employment of purgatives for the cure of this disease, we believe to be founded on a mistaken pathology; and calomel, to produce a good effect, must be given as an alterative, not as a purgative. The author's plan of administering opiates is commendable. It is true that many physicians object to their employment; but we have so often seen good effects follow their administration, that we cannot hesitate to recommend them. We would be pleased to notice this article at greater length, but are fearful of exceeding our prescribed limits.

The next article embraces a history and treatment of cholera infantum. It appears from statistics given in the volume, that this is one of the most frequent and fatal affections of childhood, being most fatal during the first year of life. This being the case, dentition is not so frequent a cause as might be supposed; though it doubtless exerts considerable influence. The other principal causes are improper aliment, the heat of summer, unhealthy location, and hereditary predisposition. Perhaps the most powerful predisposing causes are impure air and heat, for we find this disease producing the greatest ravages in the large cities, and prevailing almost exclusively during the summer months; whereas, if dentition were sufficient to produce it, we would often meet with it during the winter months, which is not the case. A disease of the importance of cholera infantum is not to be slightly passed over, and we shall therefore offer no apology for the insertion of the following extract treating of the nature of the complaint:

From the description of the anatomical lesions already given, it appears that the most characteristic and constant morbid alterations are *development* and *ulceration* of the follicular apparatus of the stomach and bowels. The mucous crypts are stated to have been much developed in the stomach only in one instance, and slightly developed in three. They were not ulcerated in any. In the small intestine they were more frequently affected, having been found numerous and distinct in two cases; distinct only in the ileum in two, and only in the duodenum in one; and as slightly developed in nine. In two only were they slightly ulcerated. Of 14 cases, therefore, in which their condition in the small intestine is mentioned, they were much developed only in two, and ulcerated in the same numbur. In the remaining cases, the alterations were slight, or observed only over a small part of the bowel, generally in the ileum or duodenum. The agminated glands were natural in 6 cases, more developed than usual, and generally reddened in 6, and not ulcerated in any. Of 12 cases, therefore, in which their condition is described, they were developed in half, and natural in the remainder. In the large intestine the crypts are stated to have been developed in all the 17 cases. In 10 of these they were ulcerated, in 4 not ulcerated, while in the remaining 3 their condition as to ulceration is not mentioned. To recapitulate: the follicles are noted as having been developed in the stomach in three cases, as ulcerated in none; in the small intestine they were numerously developed in two cases, more slightly so in twelve others, and slightly ulcerated in two; in the large intestine, they were developed in all, and of 14 in which their conditon as to ulceration is mentioned, that lesion was noted in 10. It is clear, therefore, that the follicular disease is most constant and extensive in the large intestine, less so in the small bowel, and but seldom present in the stomach.

As to *Inflammation* of the mucous membrane, we found that of 16 cases in which the state of the membrane was noted in the stomach, that lesion was present to greater or less extent, generally very slight in 6, while in 10 the tissue was pale and natural; of 16 cases in which its condition is noted in the small intestines, it was found inflamed, usually in the duodenum or ileum only, in 7, while in 9 it was noted as pale; the condition of the mucous membrane of the large intestine as to inflammation is mentioned in 15 cases, in 9 of which it was more or less inflamed throughout, in 2 the inflammation was confined to the rectum, in 4 it was slight. Inflammation was observed much the most frequently and extensively therefore in the large intestine, only half as frequently in the small intestine, and to a much smaller extent, and in a rather smaller proportion of cases in the stomach.

*Softening* existed to a greater or less extent in the stomach, in 10 out of 14 cases in which the condition of the mucous membrane as to that leston was noted; of 12 cases in which it was sought for, in the small intestines, it was present in 5, slight in all, while the membrane was natural in the other 7; of 11 cases in which it was noted in the large intestine, it was present in 9, absent in 1, while thickening existed in the remaining case. Softening existed in the stomach and large intestine in about three-fourths of the cases, and in the small intestine in rather less than half the cases.

As to the other abdominal organs, it was ascertained that the liver, which has been thought by many authors to play so great a part in the pathology of the disease, was much enlarged only in one case; and that the mesenteric glands, spleen, and kidneys were healthy. The brain, on the contrary, generally presented some injection of the membranes, and in most of the cases which proved fatal in the third stage, there was extensive disorganization of its substance from softening.

For my own part, I am disposed to believe that cholera infantum is a disease of the mucous membrane of the alimentary canal, which, beginning with morbid development of the mucous follicles or crypts, independent of *evident* inflammation, occasions first supersecretions from those organs, and after a time runs into inflammation and its results, ulceration, softening, and thickening. That it is not an inflammation in the beginning, is, it seems to me clear, from the nature of the anatomical lesions, and from the facts that the early stage is often unaccompanied by any febrile movement whatever, and is not unfrequently attended with disposition to collapse, like that which occurs in the cholera of the adult; but that it becomes an inflammation, after the development of the follicular apparatus has lasted a short time, is also, I think, apparent, from the nature of the anatomical lesions and from the circumstance that there is always more or less violent febrile reaction after the first few days.

From this it will be seen that our author lays little stress on the part the liver has been supposed to play in this disease, and in this we believe he has done good service to the profession. It has been to much the custom to lay all the blame upon the liver; it has been made the scape-goat for all the sins of the abdominal organs; hence calomel has been the alpha and the omega of treatment, much to the detriment of the profession, and the profit of charlatans. Our author has conferred a benefit upon us, by his endeavors towards a better pathology, and one for which we sincerely thank him. The course of treatment he recommends for this disease, embraces a wide range of therapeutical agents, amongst which we are rather surprised to see that he makes no mention of the alkalies and alkaline salts, which play so important a part in the practice of British physicians. We have often seen nausea allayed in the most prompt manner by carb. sodæ, when opiates, brandy, calomel, and poultices had all failed.

We omit a notice of the article on dysentery, as it presents nothing striking or original; and pass to the consideration of the section devoted to nervous diseases. These, like the diseases of the abdomen, are divided into those marked by anatomical lesions, and those which are not. Under the first head we have tubercular meningitis, cerebral congestion, hemorrhage, and acute hydrocephalus; he gives but casual mention of chronic hydrocephalus, an affection which, though rare, is certainly more frequent than hemorrhage, and perhaps as much an idiopathic complaint as ascites. In the present state of medical science, the diseases of the brain and nervous system are involved in considerable obscurity, and conclusions drawn by medical theorists are to be received with caution; but we believe our author is fully up with the latest doctrines and discoveries, and has given us a faithful abstract of the opinions of the French physicians. The article on tubercular meningitis is thorough and able, laying down faithfully all that is known of the nature and treatment of a disease almost universally fatal. Cerebral hemorrhage must be a very infrequent affection in children; and we can hardly see the necessity for the insertion of an article treating upon this subject, in a work of such limited extent as the present. We have not space to consider this class of diseases at greater length, and pass to diseases of the nervous system, unattended by appreciable anatomical lesions. These are, according to Dr. M., eclampsia or convulsions, laryngismus stridulus, contraction with rigidity, and chorea. The article on convulsions is written with care, and embodies the opinions of the highest and latest authorities. Reference is made to recently promulgated opinions of Marshal Hall, which we apprehend are not generally known to the profession in this country. We append an extract to show the importance attached by this distinguished author to the condition of the glottis in convulsions.

Dr. Hall ascribes great importance to the condition of the glottis in convulsions. He says (p. 323), in speaking of epilepsy, "The second symptom is a forcible closure of the *larynx*, and *expiratory efforts*, which suffuse the countenance, and probably congest the brain wih venous blood." At page 327, he says, "A spasmodic affection of the larynx has obviously much to do in this disease (epilepsy), as well as in the crowing inspiration or croup-like convulsion of infants; so much, indeed that I doubt whether *convulsion* would occur without closure of this organ." In describing the croup-like convul sion or laryngismus stridulus (p. 180), he says: "I must repeat the observation that the respiration is actually arrested by the closure of the larynx; aud that there are forcible expiratory efforts only, or principally, in the actual convulsion."

In a recent publication Dr. Hall says: "without closure of the larynx, extreme laryngismus, and the consequent congestion of the nervous centres, there could, I believe, be no convulsion! This closure of the larynx must be complete, in the affection under consideration, (laryngismus stridulus,) as in all others, before convulsion *can* take place." (*Braith. Ret. from Lancet*, June 12, 1847, p. 609.)

We confess that this does not seem altogether clear to us; but the whole subject is one involved in much doubt, and we take our leave of it by observing that we do not see the advantage gained in nosology, by the substitution of the term eclampsia, for the old and much more expressive cognomen, convulsions. Greek may be more intensely scientific than Latin; but for ourselves we shall not adopt the former just yet. We pass over the articles devoted to laryngismus stridulus, contraction with rigidity, and chorea.

The next division of the work is devoted to a consideration of the eruptive fevers, scarlet fever, measles, smallpox, and a section devoted to considering the necessity and propriety of revaccination. Scarlet fever is usually considered under three forms, simple, anginase, and malignant, which distinctions our author discards; substituting therefor a different classification; treating it under the heads of *regular* and grave. We plead guilty of not being able to see the improvement he has made. *Simple* and *Anginose* are expressive; and we submit that malignant is as good a term as grave. On page 468 we notice what is either a typographical error, or a great oversight on the part of the author in the statement of his cases. We have here a discussion on the use of cold affusion in this disease, and without entering into a consideration of its propriety we subjoin an extract from a letter written to Dr. M. by Dr. Corson of Pennsylvania.

"On the 16th of July, 1845, I was called to see a little girl four years and nine months old. She had been sick a day or two. The case began with vomiting. The eruption has been out since morning (now, 6 P.M.); redness the most intense, all over, that I ever saw; pulse as rapid as it could be to be counted. The mother had been alarmed during the last few hours, in consequence of delirium and jerking, which she feared was the prelude to convulsions. There was tumefaction of sub-maxillary ganglions; tongue ferred, with projecting red points; breath hot and offensive. When she found some one holding her wrist, she started from her dozing state, and being somewhat afraid of the 'doctor,' went off immediately into one of the most terrific convulsions that I ever saw. It lasted, in spite of ice to the head, or rather iced water *constantly* poured upon it, almost half an hour. I stayed with her, had her undressed and placed two nieces of mine (her mother being one) by her side. A large tub of water with cakes of ice, at least a peck, floating in it, was brought into the room, and during the *whole* night, these two persons bathed her from head to foot with the water from this tub, applying it by means of large sponges, it was to me a most painful case (independent of the convulsions), but in order to be certain that I had a case fit for the trial of the ice, I had my brother (a physician practising at Norristown, where the disease was very fatal) brought at 10 P.M., to see the case, and to say whether it was the same as those that had for a few weeks been carrying off some of the finest children in Norristown, and terror into every family. He assured me it was one of the most violent character, and that she would in all probability not live till morning. She was at this time free from convulsions, but in a muttering delirium. As I had perfect control in the case, I assured him that she should live if I could quench the fire that was burning out the vitals, by the use of ice. Not a moment did the attendants whom I had placed by her intermit their labors. Before midnight reason had returned, and her mother said she was more herself than she had been the whole day. I had gone away, but returned at sunrise, and found her cooled off perfectly. There was scarcely the least appearance of eruption, the head was cool the skin was cool, the intellect clear, and pulse moderate in frequency and force. She had been made unable to drink for many hours, and her tongue, which had been very much cut during the convulsion, was so swelled and sore, that I could obtain no view of the throat. I n w directed the mother to intermit the sponging, doing it only once in every two hours, until I returned. My return was delayed until 4 P. M., when I found that the heat of skin, frequency of pulse, eruption and delirium, had all returned. She was moving her hands as if feeling for something, slowly protruding and withdrawing the tongue, and muttering. She did not notice her mother's questions, and was apparently unconcious to all that was going on. We threw on the water, ice-cold, in the utmost profusion, and lapped cloths dipped in the water around the neck, changing them every minute or two. We poured it upon the head constantly, holding a large basin under to catch it. In one hour, reason returned. We continued it until the eruption almost disappeared, until the child shrank from it, and until she was ready to shiver with cold. I now gave her cream of tartar and jalap, directed the water to be used just as was needed to keep down the heat, and had no further trouble with her. I forgot to say that so soon as she could swallow, cold drinks and ice were kept in the mouth. She took no more medicine. The wounds in the tongue healed up kindly."

The article on measles is a good abstract of the diagnosis, complications and treatment of this disease, but presents nothing worthy of special remark. We hardly see the utility in presenting in a work upon the diseases of children, an article on smallpox; and the more especially in a work like the present, of limited extent. We think there are some other disease of more frequent comparative occurence in children than variola, concerning which our author has given us no information. We have no account in this volume of scrofula, rickets, or cyanosis; nor anything on diseases of the skin. Every young physician is liable to be called upon to give advice upon the hygienic management of children in health; and we think our author could have furnished something upon this subject that would have been more useful than a treatise on a disease elaborately discussed in all the standard works on the practice of medicine. At the close of the volume are a few pages devoted to the consideration of entozoa. His remarks on these are sensible and judicious; but present nothing new or striking.

We sabjoin one or two general remarks, by way of closing this notice. For the amount of matter it contains, this little volume is rather a good one. The arrangement is generally good, and the style in which it is written, is in the main worthy of commendation, if we except an occasional tendency to pedantry, and a display of *out-of-the-way* terms and phrases. We have already hinted that there matters contained in it, which need not in these days be inserted in a work on the diseases of childhood; and that some things are omitted, which might have been properly inserted: but in making these remarks we are not unmindful that this is a first edition, and that should it reach a second, the author will doubtles aim at making any amedment that may occur to him as being necessary.

We have thus set down what occured to us during the perusal of this volume; and we have endeavored to say our say in a spirit of fairness and impartiality; freely commending what we thought well done, and frankly pointing out what we believed could be done better. E. G. M.

*Indianapolis, October*, 1848.

